# Echocardiographic assessment of right ventricular performance in COVID-19 related acute respiratory distress syndrome: the importance of systo-diastolic interaction

**DOI:** 10.1186/s13089-024-00366-5

**Published:** 2024-05-07

**Authors:** Valentino Dammassa, Costanza Natalia Julia Colombo, Massimo Erba, Fabio Ciarrocchi, Michele Pagani, Susanna Price, Francesco Mojoli, Guido Tavazzi

**Affiliations:** 1 Department of Experimental Medicine, University of Pavia, Pavia, Italy; 2 Adult Intensive Care Unit, Royal Brompton Hospital, London, UK; 3 Anesthesia and Intensive Care, Fondazione IRCCS Policlinico San Matteo, Pavia, Italy; 4 National Heart and Lung Institute, Imperial College, London, UK; 5 Department of Clinical-Surgical, Diagnostic and Pediatric Sciences, Unit of Anesthesia and Intensive Care, University of Pavia, Pavia, Italy

**Keywords:** Echocardiography, Right ventricle, Diastolic function, Cardiac performance, Acute respiratory distress syndrome (ARDS), COVID-19

## Abstract

**Background:**

The cardiac manifestations of COVID-19 have been described in patients with acute respiratory distress syndrome (ARDS) admitted to intensive care unit (ICU). The presence and impact of right ventricular (RV) diastolic function and performance has not been studied in this population yet. We describe the prevalence of RV diastolic dysfunction, assessed by the pulmonary valve pre-ejection A wave (PV A wave), and the RV systo-diastolic interaction, using the RV total isovolumic time (t-IVT), in COVID-19 ARDS.

**Results:**

Prospective observational study enrolling patients with moderate to severe COVID-19 ARDS admitted to ICU who underwent a transthoracic echocardiogram within 24 h of ICU admission and at least a second one during the ICU stay. Respiratory, hemodynamic and biochemistry parameters were collected. 163 patients (age 61.0 ± 9.3 years, 72% males) were enrolled. 36 patients (22.1%) had RV dysfunction, 45 (27.1%) LV systolic dysfunction. 73 patients (44.7%) had PV A wave. The RV t-IVT correlated with TAPSE at ICU admission (p < 0.002; r – 0.61), presence of PV A wave (p < 0.001; r 0.78), peak inspiratory pressure (PIP) (p < 0.001; r 0.42), PEEP (p < 0.001; r 0.68), dynamic driving pressure (DDP) (p < 0.001; r 0.58), and PaO_2_/FiO_2_ ratio (p < 0.01; r − 0.35). The presence of PV A wave was associated with higher PIP (p < 0.001; r 0.45), higher PEEP (p < 0.001; r 0.56), higher DDP (p < 0.01, r 0.51), and lower PaO_2_/FiO_2_ ratio (p < 0.001; r – 0.49).

**Conclusions:**

RV t-IVT and the presence of PV A wave are non-invasive means to describe a significant RV diastolic dysfunction and may be consider descriptive signs of RV performance in COVID-19 ARDS.

## Background

The cardiac manifestations of COVID-19 have been extensively described in patients requiring intensive care unit (ICU) admission [1, 2]. In a multi-national study, first echocardiography after ICU admission revealed an abnormal cardiac function in almost one-third of critically ill COVID-19 patients with left ventricular (LV) and right ventricular (RV) systolic dysfunction observed in 23% and 22.5% of subjects, respectively [3]. Furthermore, new data are emerging about the progressive decline in left and right heart functions at 3 months after hospital discharge in patients with moderate and severe COVID-19 [4].

Both direct and indirect mechanisms of myocardial damage have been ascribed to SARS-CoV-2 [1]. If the pattern of LV systolic dysfunction resembles the one described in septic cardiomyopathy, RV involvement in critically ill COVID-19 patients appears to be multifaceted and related to ventilator parameters and presence of pulmonary embolism [3]. A particular phenotype of RV radial impairment with preserved longitudinal function has been described in severe COVID-19 acute respiratory distress syndrome (ARDS) [5]. The evaluation of RV function almost invariably relies on the assessment of systolic longitudinal function and pulmonary hemodynamics. Diastolic function, its interaction with systolic phase, and the role of isovolumetric times, although recognized as key players in the RV performance [6, 7] are usually neglected.

Herein, we describe the prevalence of RV diastolic restriction, assessed by the presence of pulmonary valve pre-ejection A wave (PV A wave) [8], and the RV systo- diastolic interaction, assessed by the RV total-isovolumic time (t-IVT) [9], in a cohort of patients admitted to ICU for COVID-19 ARDS. In addition we analyzed the relation between the RV t-IVT and the presence of the PV A wave during the ICU admission.

## Methods

We conducted a prospective observational study of patients admitted to ICU with moderate to severe COVID-19 ARDS between January 2020 and April 2021, including both the first and the second “wave” of the pandemics. SARS-CoV-2 infection was defined as positive RT-PCR from a nasopharyngeal swab and/or bronchoalveolar lavage or sputum. We enrolled patients who underwent a transthoracic echocardiogram (TTE) within 24 h of ICU admission and at least a second TTE during the ICU stay or each time a hemodynamic alteration occurred or in case of an abnormal/raising troponin level (which was measured twice a week as per internal protocol) or new ECG changes were detected. The dynamic driving pressure (DDP) was calculated as the difference between peak inspiratory pressure (PIP) and positive end-expiratory pressure (PEEP). Prior studies have established DDP as an acceptable alternative to the driving pressure [10, 11].

The TTE was performed with the patient in supine position using a Vivid iq™ (GE HealthCare) or an Affiniti 50™ (Philips) echocardiography machine with a phased array probe. A superimposed ECG was used during the echocardiographic exam and the frame rate was set at 100 frames/seconds. The measured variables were acquired at end expiration and at least three measurements (five to ten beat for patients in atrial fibrillation) were averaged for each parameter.

**Fig. 1:**
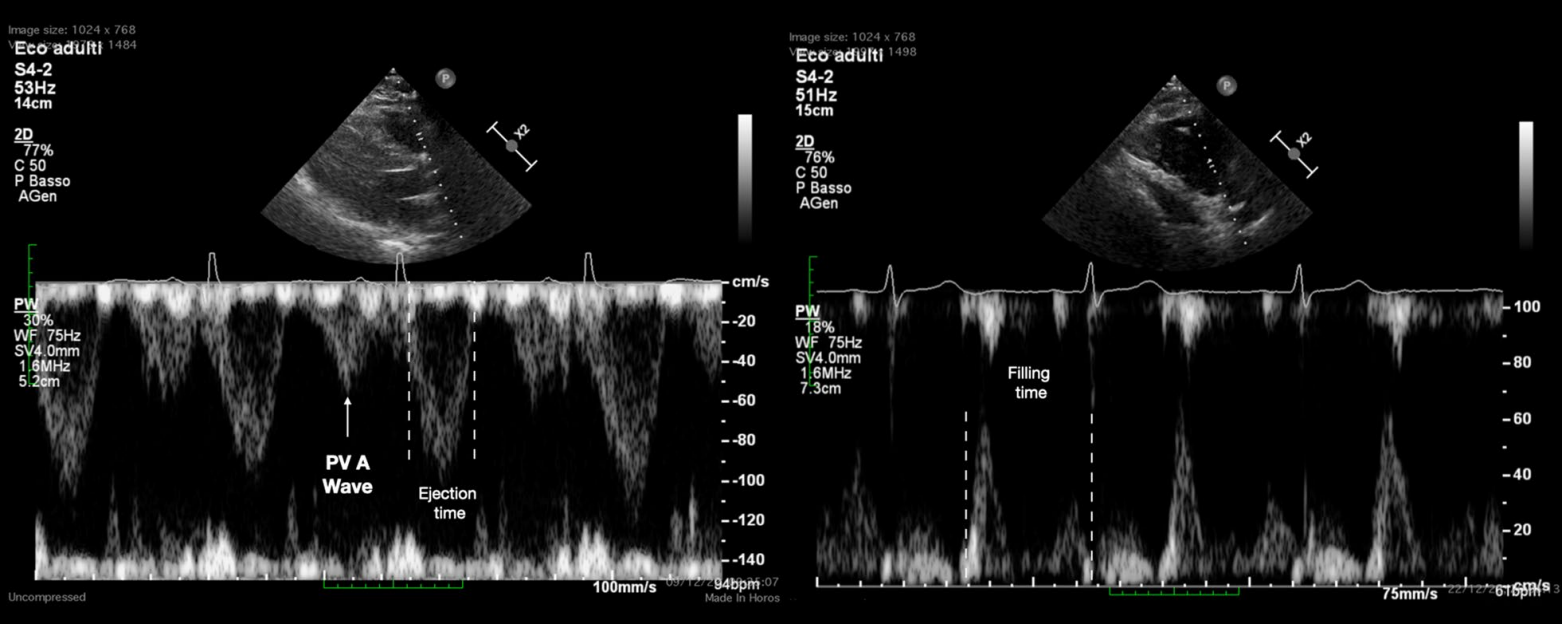
The RV total isovolumic time (RV t-IVT) represents the difference, in seconds/minute, between 60 s and the sum of total ejection time (t-ET) and total filling time (t-FT): *RV t-IVT* = *60–(t-ET* + *t-FT)*. t-ET and t-FT correspond to the heart rate-adjusted ejection time (ET) and filling time (FT) measured, respectively, as the duration of forward flow through the pulmonary valve (*left*) and from the onset of trans-tricuspid E-wave to end of trans-tricuspid A-wave (*right*). To calculate the t-ET and t-FT the following formulas are applied (where RR represents the duration in milliseconds of the R–R interval measured on the superimposed ECG): *t-ET* = [(60,000/RR) * ET]/1000 and *t-FT* = [(60,000/RR) * FT] / 1000. The pulmonary valve pre-ejection A wave (PV A wave) is identified as an anterograde flow through the pulmonary valve during atrial systole (white arrow)

LV and RV systolic dysfunction were defined respectively as a LV ejection fraction (LVEF) < 50% and a tricuspid annular plane systolic excursion (TAPSE) ≤ 16 mm. In addition to the standard European Association of Echocardiography / American Society of Echocardiography parameters [12, 13], the presence of PV A was assessed and the RV t-IVT was measured. The PV A wave was identified, using the pulsed wave Doppler, as the presence of an anterograde flow using through the pulmonary valve during the atrial systole, at superimposed ECG trace, in the parasternal RVOT outflow view or in the parasternal short-axis view (Fig. 1*—Left panel*). The systolic pulmonary artery pressure (sPAP) was estimated using the tricuspid regurgitation peak velocity and the diameter and collapsibility of the inferior vena cava [14].

The t-IVT is measured for both LV and RV and it represents the difference, expressed as seconds/minute, between 60 s and the sum of the heart rate-adjusted filling time (FT), the total filling time (t-FT), and ejection time (ET), the total ejection time (t-ET). For the RV, RV *t-IVT* = *60 – (RV t-FT* + *RV t-ET)*. The RV FT was measured from the onset of the trans-tricuspid E-wave and the end of the A-wave in the apical 4-chamber view or modified parasternal long-axis view (Fig. 1* – Right panel*). The RV ET was measured at the time interval from the onset of the forward flow across the pulmonary valve and its closure artifact in the parasternal RV outflow view or parasternal short-axis view at the level of the great vessels (Fig. 1*—*Left panel) [9]. Considering the duration (adjusted for the heart rate) of FT and ET, the t-IVT integrates in a single value both the diastolic and systolic phase of the cardiac cycle. The prolongation of the t-IVT reflects the increase in the duration of one or (more commonly) both the isovolumic phases, when the ventricle is not filling nor ejecting, throughout the cardiac cycle. Hence, the t-IVT represents a marker of electro-mechanical efficiency and ventricular performance.

The respiratory parameters (gas exchanges and ventilatory pressures) and hemodynamic data were recorded during the TTE. Biochemistry data, including markers of inflammation and cardiac injury, were also collected the day of the echocardiographic exam.

The study was approved by the local ethical committee (Fondazione Policlinico San Matteo IRCCS – 20,200,076,796–5 April 2020–). The informed consent was signed those patients who survived and was waived in those who did not survive according to the regulatory body. All the procedures were followed in accordance with the ethical standards of the responsible committee on human experimentation and with the Helsinki Declaration of 1975, as specified in the protocol.

SPSS 28.0.0 (IBM^®^ SPSS^®^ statistics) was used for data computation. Normal distribution of data was assessed with D’Agostino-Pearson test and histogram representation. We compared clinical characteristics between patients with and without evidence of RV dysfunction using the t test or Mann − Whitney U test for continuous variables and the chi-square or Fischer’s exact test for categoric variables. Analyses were performed using SPSS Categorical data were presented as percentage, while continuous data as a mean ± standard deviation or median [interquartile range] according to their distribution. Correlations were assessed with Pearson (standard deviation) or Spearman (interquartile range) tests depending on normal distribution of data and Chi square analysis was performed for nominal data. Linear regression analysis was applied to assess the predictive power of the echocardiographic indices tested and the evolution of the respiratory status.

## Results

One hundred sixty-three were enrolled having received a comprehensive TTE within 24 h of ICU admission and at least a second TTE, both of them including the Doppler assessment of PV flow for the presence of PV A wave. The mean age was 61.0 ± 9.3 years, and 72% were males. Thirty-six patients (22.1%) had RV systolic dysfunction, 45 (27.1%) had LV systolic dysfunction. Seventy-three (44.7%) patients had the PV A wave. Ninety-four patents (57.7%) had all the images to measure RV t-IVT as the trans-tricuspid inflow was included in the echocardiography clinical protocol during the second wave of the pandemics. The RV t-IVT was prolonged in 63% of patients. Thirty-five patients (21.5%) had a ratio between RV and LV end-diastolic diameter > 0.6, of whom 21 (12.9% of the overall study population) had pulmonary embolism at computed pulmonary angiography. 46 patients (28.2%) had a moderate tricuspid regurgitation, while 18 (11.0%) had a severe tricuspid regurgitation. No other hemodynamic significant valve diseases were observed. All the patients were in sinus rhythm at the time of the TTE exams. Table 1 reports the demographic features of the patients. Table 2 summarizes the clinical characteristics and the respiratory, hemodynamic, and echocardiographic parameters according to the presence of PV A wave.

**Table 1:** Clinical, respiratory, hemodynamic, and echocardiographic characteristics of the overall population

	Overall population (n = 163)
Age	61.0 ± 9.3
Diabetes mellitus	19.6% (32)
Arterial hypertension	59.5% (97)
COPD	8.6% (14)
ICU length of stay, days	22.4 ± 19.0
Death in ICU	33.1% (54)
HR, bpm	84.9 ± 19.0
MAP, mmHg	87.6 ± 15.2
Invasive mechanical ventilation	65.6% (107)
pH	7.32 ± 0.10
PaO_2_/FiO_2_ ratio, mmHg	134.9 ± 54.6
PEEP, cmH_2_O	13.4 ± 3.3
PIP, cmH_2_O	29.1 ± 4.6 (n = 107)
DDP, cmH_2_O	15.2 ± 3.6 (n = 107)
hs-TnI, ng/L	170.3 ± 518.5 (n = 160)
BNP, pg/mL	166.0 ± 347.6 (n = 157)
Noradrenaline requirement	49.7% (81)
Noradrenaline dose, mcg/kg/min	0.18 ± 0.11 (n = 81)
LVEF at ICU admission, %	50.2 ± 9.0
TAPSE at ICU admission, mm	18.3 ± 2.8
RV dilatation (RVd/LVd > 0.6)	37.4% (61)
sPAP, mmHg	39 (± 12)
TAPSE at 2nd echocardiogram, mm	17.3 ± 3.3
TAPSE variation, mm	0.0 [– 2.8 to 0.0]
RV t-IVT, s/min	11.1 ± 3.8 (n = 94)

TAPSE variation is expressed as median [interquartile range], all the other continuous variable are expressd as mean ± standard deviation

BNP, brain natriuretic peptide; COPD, chronic obstructive pulmonary disease; DDP, dynamic driving pressure; FiO_2_, fraction of inspired oxygen; HR, heart rate; hs-TnI, high-sensitivity troponin I; ICU, intensive care unit; LVd, left ventricular end-diastolic basal diameter; LVEF, left ventricular ejection fraction; MAP, mean arterial pressure; PaO_2_, partial pressure of oxygen in arterial blood; PEEP, positive end-expiratory pressure; PIP, peak inspiratory pressure; PV A wave, pulmonary valve pre-ejection A wave; TAPSE, tricuspid annular plane systolic excursion; RVd, right ventricular end-diastolic basal diameter; sPAP, systolic pulmonary artery pressure; RV t-IVT, right ventricular total isovolumic time

**Table 2:** Clinical, respiratory, hemodynamic, and echocardiographic characteristics and parameters according to the presence of PV A wave

	No PV A wave (n = 90)	Presence of PV A wave (n = 73)	p-value
Age	61.0 ± 9.2	62.0 ± 10.6	0.890
Diabetes mellitus	20.0% (18)	19.2% (14)	0.786
Arterial hypertension	50.0% (45)	71.3% (52)	0.687
COPD	6.7% (6)	10.9% (8)	0.658
ICU length of stay, days	20.4 ± 18.7	25.0 ± 19.3	0.121
Death in ICU	26.7% (24)	41.1% (30)	0.138
HR, bpm	84.1 ± 18.2	85.8 ± 20.0	0.572
MAP, mmHg	87.5 ± 13.8	87.7 ± 16.9	0.940
Invasive mechanical ventilation	52.2% (47)	82.2% (60)	< 0.001
pH	7.33 ± 0.10	7.31 ± 0.10	0.234
PaO_2_/FiO_2_ ratio, mmHg	158.9 ± 52.7	105.5 ± 41.1	< 0.001
PEEP, cmH_2_O	12.0 ± 2.7	15.1 ± 3.3	< 0.001
PIP, cmH_2_O	26.4 ± 4.6 (n = 47)	30.0 ± 4.5 (n = 60)	< 0.01
hs-TnI, ng/L	84.8 ± 192.6 (n = 88)	274.7 ± 732.4 (n = 72)	0.020
BNP, pg/mL	144.4 ± 274.4 (n = 86)	192.2 ± 220.2 (n = 71)	0.392
Noradrenaline requirement	38.9% (35)	63.0% (46)	0.002
Noradrenaline dose, mcg/kg/min	0.15 ± 0.09 (n = 35)	0.19 ± 0.12 (n = 46)	0.073
LVEF at ICU admission, %	50.2 ± 10.2	50.2 ± 7.4	0.964
TAPSE at ICU admission, mm	18.3 ± 2.6	18.4 ± 3.1	0.939
RV dilatation (RVd/LVd > 0.6)	33.3% (30)	42.5% (31)	0.228
sPAP, mmHg	35 (± 6)	44 (± 8)	< 0.001
TAPSE at 2nd echocardiogram, mm	18.4 ± 2.9	16.0 ± 3.2	< 0.001
TAPSE variation, mm	0.0 [0.0–0.0]	– 2.0 [– 4.0 to – 1.0]	< 0.001
RV t-IVT, s/min	8.2 ± 1.9 (n = 46)	14.0 ± 3.0 (n = 48)	< 0.001

TAPSE variation is expressed as median [interquartile range], all the other continuous variable are expressd as mean ± standard deviation

BNP, brain natriuretic peptide; COPD, chronic obstructive pulmonary disease; DDP, dynamic driving pressure; FiO_2_, fraction of inspired oxygen; HR, heart rate; hs-TnI, high-sensitivity troponin I; ICU, intensive care unit; LVd, left ventricular end-diastolic basal diameter; LVEF, left ventricular ejection fraction; MAP, mean arterial pressure; PaO_2_, partial pressure of oxygen in arterial blood; PEEP, positive end-expiratory pressure; PIP, peak inspiratory pressure; PV A wave, pulmonary valve pre-ejection A wave; TAPSE, tricuspid annular plane systolic excursion; RVd, right ventricular end-diastolic basal diameter; sPAP, systolic pulmonary artery pressure; RV t-IVT, right ventricular total isovolumic time

There was no difference in respiratory comorbidities between patients with RV dysfunction (5% versus 7%, p 0.681), RV t-IVT prolongation (3% versus 4.2%, p 0.789) and PV A wave (3% versus 2.8%, p 0.768). There was no statistically significant difference in the incidence of acute kidney injury requiring renal replacement therapy in patients with RV dysfunction (6.6%) compared with those without RV dysfunction (7.4%, p = 0.47).

The patients who required the invasive mechanical ventilation (107, 65.7%) had more frequently the PV A wave (p < 0.001) and a prolonged RV t-IVT (p = 0.009). The differences between intubated and non-intubated patients are shown in Table 3.

**Table 3:** Differences between intubated and non- intubated patients at the time of first echocardiogram

	Invasive mechanical ventilation—intubated (n = 107)	Non-invasive ventilation (n = 56)	p-value
Age	62 ± 9.1	60 ± 11.75	0.49
BSA, m^2^	2.02 ± 0.28	1.97 ± 0.18	0.13
PaO_2_/FiO_2_ ratio, mmHg	129 ± 57.96	143 ± 41.89	0.13
PEEP, cmH_2_O	14.6 ± 3.05	11.38 ± 2.44	0.22
PIP, cmH_2_O	29.06 ± 4.33	N/a	–
DDP, cmH_2_O	14.46 ± 3.05	N/a	–
LVEF, %	50 .74 ± 8.18	50.88 ± 10.35	0.5
RV t-IVT (s/min) at ICU admission	10.36 ± 2.93	12.25 ± 4.49	< 0.001
TAPSE (mm) at ICU admission	18.04 ± 2.91	18.92 ± 2.50	0.66
PV A wave	12	64	< 0.001*
RV t-IVT (s/min) at 2nd echocardiogram	11.11 ± 3.9	8. 15 ± 1.15	< 0.001
TAPSE (mm) at 2nd echocardiogram	17.98 ± 2.54	18.24 ± 2.4	0.58

DDP, dynamic driving pressure; FiO_2_, fraction of inspired oxygen; ICU, intensive care unit; LVEF, left ventricular ejection fraction; PaO_2_, partial pressure of oxygen in arterial blood; PEEP, positive end-expiratory pressure; PIP, peak inspiratory pressure; PV A wave, pulmonary valve pre-ejection A wave; TAPSE, tricuspid annular plane systolic excursion; RV t-IVT, right ventricular total isovolumic time

^*^Chi square analysis was run

The RV t-IVT correlated with the TAPSE at ICU admission (p < 0.002; r – 0.61), the TAPSE at the second TTE (p < 0.001; r – 0.44), the presence of PV A wave (p < 0.001; r 0.78), PIP (p < 0.001; r 0.42), PEEP (p < 0.001; r 0.68), DDP (p < 0.001; r 0.58), and the ratio between partial pressure of oxygen in arterial blood and fraction of inspired oxygen (PaO_2_/FiO_2_ ratio) (p < 0.01; r – 0.35). Additionally, RV t-IVT had a positive correlation with serum creatinine at admission (p 0.02; r 0.41). The RV t-IVT did not correlate with the mean arterial pressure (p = 0.56), high-sensitivity troponin I (p = 0.68), brain natriuretic peptide (p = 0.72), or heart rate (p = 0.07). The TAPSE did not correlate with PaO_2_/FiO_2_ ratio (p = 0.46) or PEEP (p = 0.65).

The presence of the PV A wave at first point was associated with higher peak inspiratory pressure (PIP) (p < 0.01; r 0.45), PaCO_2_ (p 0.01; r 0.56), higher PEEP (p < 0.001; r 0.56), higher DDP (p < 0.01, r 0.51) and lower PaO_2_/FiO_2_ ratio (p < 0.001; r – 0.49). There was no statistically significant difference in RV and LV systolic function at ICU admission between patients with and without the PV A wave (p = 0.939 and 0.964, respectively). Estimated sPAP was higher in patients with the PV A wave (p < 0.001). The presence of the PV A wave demonstrated a trend towards statistical significance with RV/LV basal diameter (p = 0.053) (Table 2).

At the linear regression analysis only the RV t-IVT predicted the worsening of TAPSE (p < 0.001; r 0.65; 95% CI [– 0.59 to – 0.19]).

**Fig. 2:**
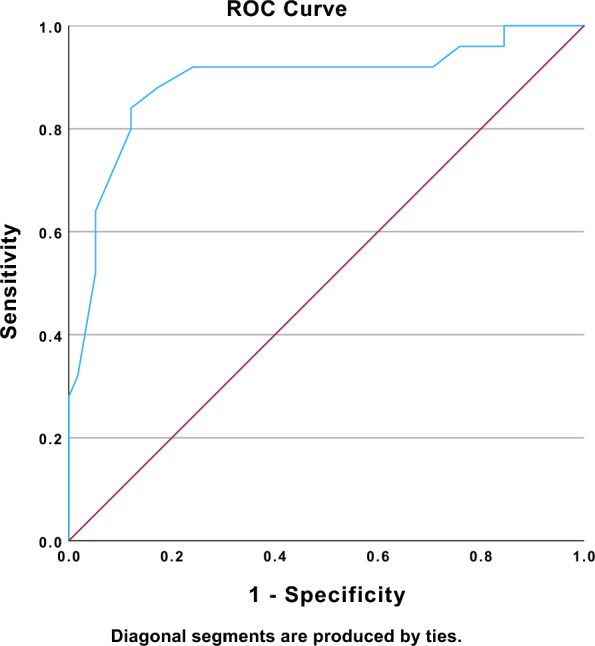
Area under the receiver operating characteristic (ROC) curve of the RV t-IVT in predicting a worsen ventilation status

**Fig. 3:**
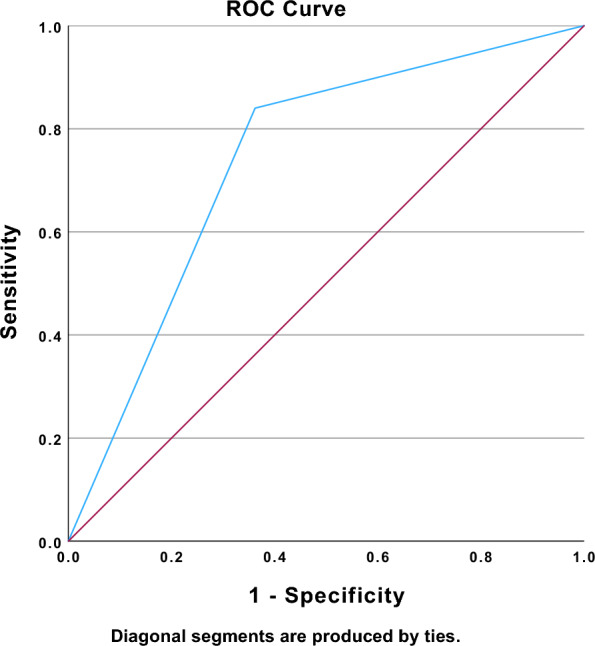
Area under the receiver operating characteristic (ROC) curve of the presence of PV A wave in predicting a worsen ventilation status

The presence of PV A wave and RV t-IVT predicted the worsening of ventilation status from non-invasive to invasive ventilation (respectively, p < 0.01 r 0.449 and p < 0.001, r 0.684—Figs. 2 and 3); also TAPSE value at the time of ICU admission was associated with upscale to mechanical ventilation but with a remarkably low level of correlation coefficient (p 0.023, r 0.153). The area under the ROC curve for the prediction model was respectively: RV t-IVT 0.891 (Fig. 2), PV A wave 0.739 (Fig. 3), and TAPSE 0.499.

## Discussion

In the evaluation of RV performance of patients with respiratory failure, the RV t-IVT and the presence of the PV A wave may be included on top of the commonly used parameter to comprehensively assess the effect of heart lung interaction. The increased RV afterload related to both ARDS pathophysiology and positive-pressure ventilation may significantly alter this interaction resulting in RV dysfunction and restrictive RV compliance.

The RV systolic dysfunction and increased ventilatory pressures are both related with adverse outcome in critically ill patients undertaking mechanical ventilation either in ARDS related and not related to COVID-19 [3, 15]. The presence of RV dysfunction has been already reported in patients with COVID-19 ARDS requiring ICU admission [3]. However, the main focus of the studies describing RV function and dysfunction in patients with ARDS, before and during the COVID-19 pandemics, was the presence of RV dilatation and systolic impairment (mainly based on TAPSE [3, 15] and less frequently on radial function [5]) describing a more advanced stage of RV failure.

The t-IVT is an index of electromechanical efficiency representing the amount of time spent by the ventricle not ejecting nor filling over a minute. The normal t-IVT for the RV is 7.0 ± 1.1 s/minute, and its value increases significantly with the age [9]. The RV t-IVT is shorter compared to the LV, which has a normal t-IVT < 14.0 s/minute. This difference is consistent with the RV physiology explored on the pressure/volume loop analysis, and it is mainly related to RV-pulmonary vascular tree interaction, leading to shorter isovolumetric times for the RV [16]. Although there are not any prior studies on RV t-IVT in cardiac disease, LV t-IVT has been reported as one the most accurate index of electro-mechanical performance in patients with coronary artery disease and dilated cardiomyopathy [17–19]. Furthermore, the LV t-IVT demonstrated a higher sensitivity as compared to LVEF to frame the best hemodynamic profile, by titrating heart rate according to it, in two small series of patients with cardiogenic shock and post-cardiotomy hemodynamic instability [20, 21]. Generally, the interaction between time intervals and perfusion alteration, diastolic/strain are well documented since many years in cardiovascular and respiratory patients [22–25]. Once more, the integration of them into a comprehensive evaluation of systo-distolic interaction has never been reported before. The RV t-IVT, representing the integration of the whole cardiac cycle, may be interpreted as the ventricular performance, although this concept must be validated in wider and different populations, such as chronic pulmonary hypertension patients.

The increase in RV size is usually considered the landmark of RV dysfunction. The RV dilatation is the compensatory response to the afterload increase according to the Frank-Starling law. The leftward septal bowing is usually observed in RV dilatation and reflects a prolonged contraction time of the RV with respect to LV contraction time [26]. A prolongation of the RV longitudinal contraction leading to a restrictive RV pattern, assessed by echocardiography, has already been reported in patients with ARDS [8]. The main mechanism appears to be the increased wall tension with the RV contraction that continues while the LV is already in its diastolic phase [27]. This phenomenon may be potentially unmasked or exacerbated by hypoxemia resulting in a mismatch between oxygen demand and supply. This mechanism underlies the potential prolongation of the RV t-IVT as it usually happens at the expense of the diastolic filling phase [17] which in this setting may potentially in turn contribute to the onset of the RV restrictive pattern. On top of hypoxemia, the pathophysiology of the RV dysfunction in ARDS is related mostly to the changes occurring at the capillary level including endothelial dysfunction, pulmonary microvascular occlusion, release of pulmonary vasoactive mediators, and alterations in pulmonary vasomotor tone [28]. These mechanisms may result in increased RV afterload and RV-pulmonary arterial decoupling.

The PV A wave represents the presence of flow across pulmonary valve generated by atrial contraction in a poorly compliant RV. This echocardiographic finding was found to be associated with diastolic pulmonary pressure greater than 20 mmHg in patients with complete repair of tetralogy of Fallot [29]. The PV A wave was also identified in ARDS patients with high ventilatory pressure and hypercapni a[8] Since acute hypoxemia may induce ventricular diastolic dysfunction [30], it may be speculated that the correlation between the severity of hypoxemia (expressed by the PaO_2_/FiO_2_ ratio) and the presence of PV A wave may be related to an oxygen supply–demand mismatch entailing a worse myocardial perfusion, but this has yet to be demonstrated.

The correlation between RV electro-mechanical synchrony (prolonged t-IVT) and RV restrictive pattern with ventilatory pressures may be interpreted as a casual effect of the latter or as higher acuity of patients with severe respiratory compromise. Similarly, the regression analysis should be contextualized and further investigated. It seems more likely, considering the pathophysiology of such population, that the prolongation of RV t-IVT and the presence of restrictive compliance may play as marker of severity of the heart–lung interaction.

There are many limitation to be acknowledged. The first one is the relatively small sample size. The main limitation of our study is the potential selection bias. The patients receiving the second TTE were those who had clinical reasons driving at least to a second examination (either elevation of high-sensitivity troponin, new ECG changes or hemodynamic deteriorations). Not all patients had an echocardiographic exam including the tricuspid inflow measurement with pulsed wave Doppler (due to internal protocol during the first wave of the pandemics), therefore the logistic model had to be run two times excluding those who did not have RV t-IVT. This may have influenced the results of the regression model which included RV t-IVT. The aim of the study was the definition of RV function and its changes over time, therefore only parameters fulfilling this aim have been analyzed.

## Conclusions

RV t-IVT and PV A wave not only represent a useful non-invasive means to describe significant RV diastolic dysfunction but may be also consider as descriptive signs of the underlying RV systo-diastolic interaction pathophysiology in response to increased afterload. The role of RV t-IVT value at ICU admission in predicting the worsening of RV systolic function may hasten the early identification of those patients requiring a careful monitoring of heart–lung interaction and titration of ventilatory support.

## Data Availability

The datasets used and/or analysed during the current study are available from the corresponding author on reasonable request.

## Abbreviations

**A**RDS: Acute respiratory distress syndrome
BNP: Brain natriuretic peptide
COPD: Chronic obstructive pulmonary disease
DDP: Dynamic driving pressure
ET: Ejection time
FT: Filling time
FiO_2_: Fraction of inspired oxygen
HR: Heart rate
hs-TnI: High-sensitivity troponin I
ICU: Intensive care unit
LV: Left ventricle/ventricular
LVEDA: Left ventricular end-diastolic area
LVEF: Left ventricular ejection fraction
MAP: Mean arterial pressure
PaO_2_: Partial pressure of oxygen in arterial blood
PEEP: Positive end-expiratory pressure
PIP: Peak inspiratory pressure
PV A wave: Pulmonary valve pre-ejection A wave
RT-PCR: Reverse transcription polymerase chain reaction
RV: Right ventricle/ventricular
RVEDA: Right ventricular end-diastolic area
sPAP: Systolic pulmonary artery pressure
TAPSE: Tricuspid annular plane systolic excursion
t-ET: Total ejection time
t-FT: Total filling time
t-IVT: Total isovolumic time
TTE: Transthoracic echocardiogram
